# Patterns of chemotherapy-associated toxicity and supportive care in US oncology practice: a nationwide prospective cohort study

**DOI:** 10.1002/cam4.200

**Published:** 2014-02-17

**Authors:** Eva Culakova, Ramya Thota, Marek S Poniewierski, Nicole M Kuderer, Adane F Wogu, David C Dale, Jeffrey Crawford, Gary H Lyman

**Affiliations:** 1 Duke UniversityDurham, North Carolina; 2 Vanderbilt UniversityNashville, Tennessee; 3 University of WashingtonSeattle, Washington; 4 Fred Hutchinson Cancer Research CenterSeattle, Washington

**Keywords:** Chemotherapy, infection, neutropenia, toxicity

## Abstract

Neutropenic complications remain an important dose-limiting toxicity of cancer chemotherapy-associated with considerable morbidity, mortality, and cost. Risk of the initial neutropenic event is greatest during the first cycle. The purpose of this study was to better understand timing of neutropenic events in relation to delivered chemotherapy dose intensity and utilization of supportive care during cancer treatment. A prospective cohort study of adult patients with solid tumors or lymphoma initiating chemotherapy was conducted at 115 randomly selected US practice sites between 2002 and 2006. Chemotherapy-associated toxicities were captured in up to four treatment cycles including severe neutropenia, febrile neutropenia, and infection. Documented interventions included colony-stimulating factor (CSF), antibiotics use, and reductions in chemotherapy relative dose intensity (RDI). A total of 3638 patients with breast (39.7%), lung (23.7%), colorectal (13.6%), ovarian (8.3%) cancers, or lymphoma (14.7%) were eligible for this analysis. The majority of neutropenic and infection events occurred in the first cycle. A significant inverse relationship was observed between reductions in neutropenic and infectious events and increased utilization of measures to reduce these complications in subsequent cycles. More than 60% of patients with stage IV solid tumors underwent reductions in RDI. Patients with lymphoma and stage I–III solid tumors had less dose reductions while receiving more prophylactic CSFs. Approximately, 15% of patients received prophylactic antibiotics. While the risk of neutropenic complications remains greatest during the initial cycle of chemotherapy, subsequently instituted clinical measures in efforts to reduce the risk of these events vary with cancer type and stage.

## Introduction

Neutropenic complications remain the main dose-limiting toxicity of cancer chemotherapy treatment and are associated with considerable morbidity and mortality 1. Febrile neutropenia (FN) represents an oncologic emergency 2. Moreover, episodes of FN are accompanied by considerable costs related to hospitalization, additional outpatient care, and substantial economic and psychological burdens on patients and their caregivers 3–6. Prior studies have shown that the risk of initial neutropenic events is greatest in the first cycle when most patients are receiving full-dose chemotherapy reporting 50–75% of initial neutropenic complications occurring within the first cycle 7–11. However, patients who have experienced an initial neutropenic event are at increased risk for additional neutropenic events during the subsequent treatment 12.

Importantly, neutropenic events such as FN, severe neutropenia (SN), or infection in the initial cycle(s) of chemotherapy frequently generate a subsequent response from clinicians to reduce the risk of repeated events including one or more of the following measures: chemotherapy dose reductions or treatment delays; use of prophylactic colony-stimulating factors (CSFs); addition of prophylactic oral antibiotics. While these measures may reduce the risk of neutropenic complications including FN in subsequent cycles 7, without such interventions, the risk for neutropenia remains high throughout the period of chemotherapy treatment 13. Nevertheless, patterns of supportive measures during the first and subsequent cycles of chemotherapy as well as patterns of associated toxicities in routine community clinical practice are largely unknown. Furthermore, the influence of targeted preventive strategies, implemented early in the course of chemotherapy, on reducing the risk of neutropenic events has not been extensively studied outside of randomized control trials (RCTs). This large, nationwide prospective cohort study was conducted to better assess neutropenic complications and to understand their influence on the patterns of supportive care and chemotherapy delivery in patients receiving cancer chemotherapy in community practice.

## Material and Methods

### Study design

A prospective cohort study was conducted to evaluate the chemotherapy-related toxicities and supportive care in a community oncology setting. Patients were accrued at 115 US randomly selected practice sites (community practices or academic cancer centers). The sites were distributed within all four geographic regions with 37 located in Central US, 22 in Northeast, 34 in South, and 22 in West Coast. The study was approved by the Institutional Review Board and all patients signed an informed consent prior to the data collection. To avoid selection bias, sites were required to enroll consecutive eligible patients who were at the initiation of their new chemotherapy regimen. The choice of chemotherapy regimen as well as course of the entire treatment was at the discretion of the treating oncologist with no specific treatment intervention required by the study. Data were collected during the first four cycles of chemotherapy and the only constraint added by the study to the usual care was that patients had to be willing to return for midcycle (nadir) visits during the four cycles. Neither the investigators nor the funding agency had direct contact with the participating sites. An independent clinical research organization coordinated the data collection process. The data analyses, reporting, and interpretation were performed at the Study Coordinating Center independent of the funding agency.

### Patient selection

Patients with cancer eligible for inclusion were adults (≥18 years) starting a new chemotherapy regimen with a minimum life expectancy of at least 3 months planning to receive at least four cycles of myelosuppressive treatment. Prior chemotherapy and concurrent radiation therapy were permitted. Patients with myeloma, leukemia, human immunodeficiency virus, history of any stem cell transplantation, or those receiving concurrent myelosuppressive drugs for medical conditions other than cancer were excluded. Additionally, patients participating in blinded RCTs, as well as those with active infection were not eligible. Study patients with breast, lung, colorectal, or ovarian cancer or lymphoma who had available toxicity data for at least the first cycle of chemotherapy were included in the analysis.

### Study variables

Data were collected for each patient at baseline prior to the treatment and at the beginning and midpoint of each cycle of the first four cycles of chemotherapy. Baseline demographic and clinical variables included age, gender, ethnicity, performance status, body surface area (BSA), cancer stage, comorbidities, prior chemotherapy treatment, and planned chemotherapy treatment. In addition, data on chemotherapy drugs, schedule and dosing information, routine laboratory tests, and adverse events were collected at every cycle. As the information about adverse events during the prior cycle was collected at the beginning of the next cycle, some data from the last collected cycle, cycle 4, about adverse events, or treatment toxicities including FN, infection, and fever were incomplete. Clinical and administrative reasons for early termination were gathered. Administrative reasons for patients dropping out from the study related to the study protocol such as requirements for nadir laboratory results and were not necessarily related to the cessation of chemotherapy.

The standard dose and schedule for utilized regimens were estimated based on the data available from RCTs. Dose intensity (DI) was defined as the amount of drug per unit of time, per m^2^ of BSA. Relative dose intensity (RDI) was defined as the ratio of either planned DI to the standard DI (planned RDI) or actual received DI to the standard DI (actual RDI). Assuming that neutropenic events within a chemotherapy cycle might be influenced by recovery time from a prior cycle and the actual dose for the cycle, the current cycle RDI was investigated within this analysis. For the first cycle, planned RDI was substituted as the current cycle RDI. For cycles 2–4, the RDI was calculated based on the length of the previous cycle and the dose given within the current cycle and compared to the standard. For each regimen, the planned, actual, and current cycle RDIs were first calculated for each drug separately and then averaged across all myelosuppressive agents given.

### Study outcomes

The primary end points of the study were chemotherapy-associated neutropenic events, specifically FN (fever/infection and absolute neutrophil count [ANC] < 1000/mm^3^), SN (ANC < 500/mm^3^), as well as documented infection or fever. Secondary end points included interventions to reduce the chemotherapy-associated toxicities, such as use of CSF and antibiotics, and reductions in chemotherapy RDI. Primary CSF prophylaxis was defined as CSF use planned at the beginning of the first cycle or before a neutropenic event within the first cycle, whereas secondary CSF prophylaxis was defined as CSF prescribed within the initiation of later cycles.

### Statistical methods

This prospective observational study was designed to describe patterns of care with the analysis primarily descriptive in nature. Proportions were presented for all relevant clinical categorical variables. The continuous variables were evaluated using standard measures of central tendency and variability summarized via descriptive statistics such as mean, median, standard deviation, and/or standard error. The proportion of patients with neutropenic events and the proportion of patients receiving supporting interventions were calculated by cycle and cumulatively across all cycles. The results are presented for all patients as well as stratified by three relevant clinical subgroups (lymphoma, patients with early-stage solid tumors, and patients with metastatic solid tumors). SAS software (version 9.3; SAS Institute Inc, Cary, NC) was used to analyze the data.

## Results

### Study participants

This prospective cohort study was conducted within the years 2002 and 2006 and enrolled a total of 4458 patients with cancer and most (94.3%) treated in community practices. This analysis is focused on 3638 patients with breast (*n* = 1443, 39.7%), lung (*n* = 863, 23.7%), colorectal (*n* = 495, 13.6%), ovarian (*n* = 303, 8.3%) cancers, and lymphoma (*n* = 534, 14.7%). The mean and median ages of patients were 60 years (standard deviation 13 years) with 38.0% of age 65 or older. The majority of patients were Caucasians (84.6%) with African–Americans constituting 10.3%. The most common evaluated comorbidities were history of anemia (16.4%), diabetes (11.2%), chronic lung disease (8.7%), and cardiovascular disease (5.6%). Among the 3104 patients with solid tumors, approximately two-thirds had stage I–III disease (*n* = 2022, 65.1%) and one-third stage IV disease (*n* = 1050, 33.8%), while for 32 patients (1.0%), the stage was unknown. Table 1 summarizes the baseline patient, disease, and treatment characteristics of the study population.

**Table 1:** Baseline patient characteristics.

	All Patients	Lymphoma	Solid tumor stage I–III	Solid tumor stage IV
Characteristics	*n* (% of 3638)	*n* (% of 534)	*n* (% of 2022)	*n* (% of 1050)
Age (years)				
<50	824 (22.6)	117 (21.9)	558 (27.6)	142 (13.5)
50–64	1430 (39.3)	158 (29.6)	854 (42.2)	405 (38.6)
65–69	465 (12.8)	68 (12.7)	212 (10.5)	184 (17.5)
70–74	409 (11.2)	68 (12.7)	192 (9.5)	144 (13.7)
≥75	510 (14.0)	123 (23.0)	206 (10.2)	175 (16.7)
Race				
Caucasian	3076 (84.6)	475 (89)	1682 (83.2)	890 (84.8)
African–American	373 (10.3)	36 (6.7)	215 (10.6)	120 (11.4)
Other	189 (5.2)	23 (4.3)	125 (6.2)	40 (3.8)
ECOG PS				
0	2047 (56.3)	285 (53.4)	1365 (67.5)	379 (36.1)
1	1297 (35.7)	203 (38)	578 (28.6)	505 (48.1)
≥2	294 (8.1)	46 (8.6)	79 (3.9)	166 (15.8)
Baseline BSA				
≤2 m^2^	2599 (71.4)	321 (60.1)	1520 (75.2)	738 (70.3)
>2 m^2^	1039 (28.6)	213 (39.9)	502 (24.8)	312 (29.7)
Medical history				
Prior chemotherapy	847 (23.3)	94 (17.6)	289 (14.3)	457 (43.5)
Recent surgery	1205 (33.1)	168 (31.5)	837 (41.4)	192 (18.3)
Diabetes	409 (11.2)	64 (12)	196 (9.7)	142 (13.5)
Cardiac disease	205 (5.6)	42 (7.9)	87 (4.3)	75 (7.1)
Lung disease	317 (8.7)	26 (4.9)	145 (7.2)	144 (13.7)
History of anemia	598 (16.4)	97 (18.2)	249 (12.3)	246 (23.4)
Chemotherapy treatment				
Anthracyclines	1438 (39.5)	368 (68.9)	986 (48.8)	74 (7.0)
Taxanes	1048 (28.8)	1 (0.2)	605 (29.9)	427 (40.7)
Platinums	1150 (31.6)	11 (2.1)	558 (27.6)	562 (53.5)
Two or more myelosuppressive drugs	2963 (81.4)	425 (79.6)	1728 (85.5)	780 (74.3)

ECOG PS, eastern cooperative group performance status; BSA, body surface area.

### Neutropenic and infectious events

**Figure 1:**
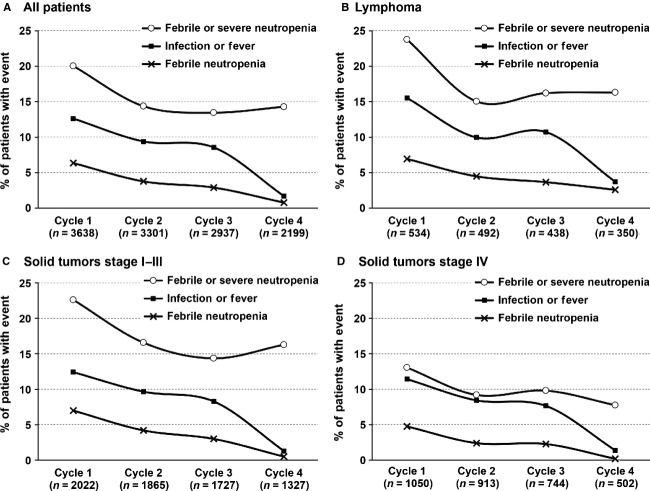
Cycle-specific neutropenic and infection events during chemotherapy treatment cycles for all patients (A) and among lymphoma (B), early stage (C), or late stage solid tumor (D) patients.

The highest occurrence of neutropenic and infectious events occurred in cycle 1 (Fig. 1) with a substantial decrease seen in subsequent cycles. FN events decreased from 6.4% in cycle 1 to 3.8% and 2.9% in cycles 2 and 3, respectively. In comparison, compound events of febrile and/or severe neutropenia (FN/SN) decreased from 20% in cycle 1 to 14% in cycle 2 and stayed approximately stable at this lower level with 13.5% and 14.3% in cycles 3 and 4, respectively. This decreasing trend of neutropenic events in subsequent cycles was uniformly noted in patients with lymphoma, early-stage solid tumors, or stage IV solid tumors. However, the incidence of FN/SN events in each cycle was influenced by cancer type and disease stage, with first cycle neutropenic event rates of 23.8% in lymphoma, 22.6% in stage I–III solid tumors, and 13.1% in stage IV solid tumors. Similarly, during the subsequent cycles (2, 3, and 4), lower occurrences of FN/SN were observed in patients with stage IV solid tumors (9.2%, 9.8%, 7.8%) compared to patients with stage I–III solid tumors (16.6%, 14.4%, 16.3%) or lymphoma (15.0%, 16.2%, 16.3%). Febrile and infectious events followed a comparable trend.

### Supportive care measures

**Figure 2:**
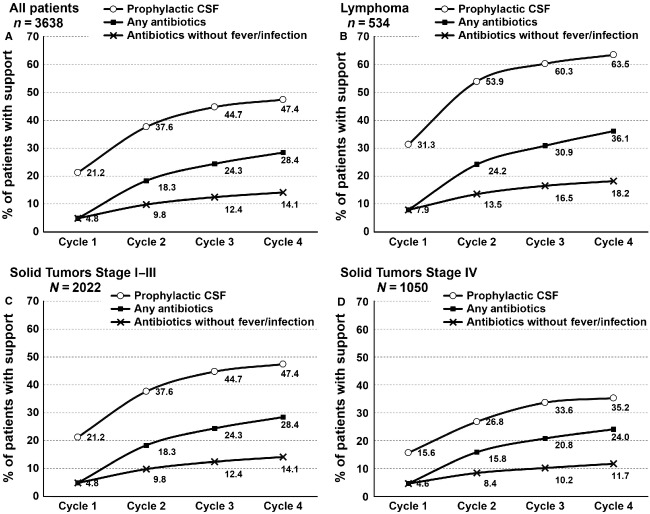
Cumulative use of prophylactic growth factor and antibiotics initiated by a specific cycle for all patients (A) and among lymphoma (B), early stage (C), or late stage solid tumor (D) patients.

The cumulative use of prophylactic myeloid growth factors and antibiotic use to reduce chemotherapy-related toxicities increased from cycle 1 through cycle 4 (Fig. 2). Prophylactic CSF use more than doubled from cycle 1 to cycle 4 (21.2% in cycle 1; 47.4% overall). A similar increase was noted in antibiotic use in the first and subsequent cycles (4.8% in cycle 1; 24.8% overall). Approximately 15% of patients received antibiotics in the absence of reported fever or infection. It was noted that prophylactic use of CSF and antibiotics use was higher in lymphoma patients compared to those with solid tumors (Fig. 2).

### RDI

A substantial proportion of patients in each cancer group underwent major reductions in chemotherapy RDI, with more than half of patients with lymphoma (55.8%) or stage I–III solid tumor (51.1%) and two-thirds with stage IV solid tumor (67.3%) receiving RDI < 90% in one or more cycles. Likewise, more than one-third of patients with lymphoma (38.9%) and about a third of patients with stage I–III solid tumors (32.7%) and more than half (51.7%) of patients with stage IV solid tumors received overall actual RDI < 85%. Reductions in RDI varied with cancer type and stage, with the reduction in DI most commonly seen in patients with stage IV solid tumors. Among patients with stage IV disease, planned RDI < 85% of standard was observed in 47.4% of ovarian, 32.8% breast, 32.8% colorectal, 30.5% small cell lung, and 28.7% non-small cell lung cancer patients. However, also 22.4% lymphoma patients and 13.4% of patients with early-stage breast cancer had planned RDI < 85%. The detailed information on the reduced RDI by cancer type and stage is presented in Table 2. Additional factors influenced both planned and received RDI. Approximately half (50.2%) of patients with age ≥65 years and 52.5% of patients with prior history of chemotherapy received actual RDI < 85% in contrast to 31.8% of younger and 34.9% of first-line treatment patients. Simultaneously 42.3% of patients with age ≥ 65 and 40.0% of patients with prior chemotherapy received CSF support compared to about 50% use among younger or chemotherapy naïve patients.

**Table 2:** Patients with reduced RDI by cancer type and stage (percent of patients).

Cancer type (*n*,% of patients within the group)	Planned RDI < 85%	Overall actual RDI < 85%	RDI < 85% for one or more cycles	Planned RDI < 90%	Overall actual RDI < 90%	RDI < 90% for one or more cycles
Lymphoma (*n* = 459)	22.4	38.9	48.4	30.1	48.0	55.8
Stage I–III (*n* = 1946)	21.6	32.7	43.3	27.0	41.4	51.1
Breast (*n* = 1176, 60%)	13.4	22.2	32.5	17.0	30.5	40.0
Non-small cell lung (*n* = 269, 14%)	42.0	63.0	63.9	52.8	71.3	75.1
Small cell lung (*n* = 71, 4%)	33.8	66.2	69.0	45.1	80.3	78.9
Colorectal (*n* = 238, 12%)	21.0	41.7	47.5	24.8	47.2	52.5
Ovarian (*n* = 192, 10%)	39.6	59.4	66.1	47.9	67.2	74.0
Stage IV (*n* = 961)	32.7	51.7	60.2	41.7	63.3	67.3
Breast (*n* = 204, 21%)	32.8	53.2	63.2	39.7	62.1	69.6
Non-small cell lung (*n* = 344, 36%)	30.5	58.8	59.3	41.0	66.5	66.6
Small cell lung (*n* = 108, 11%)	28.7	54.6	52.8	45.4	61.1	63.0
Colorectal (*n* = 229, 24%)	32.8	50.7	59.0	37.6	58.7	65.1
Ovarian (*n* = 76, 8%)	47.4	63.2	71.1	57.9	69.7	77.6

RDI, relative dose intensity.

### First cycle and subsequent cycle events

**Figure 3:**
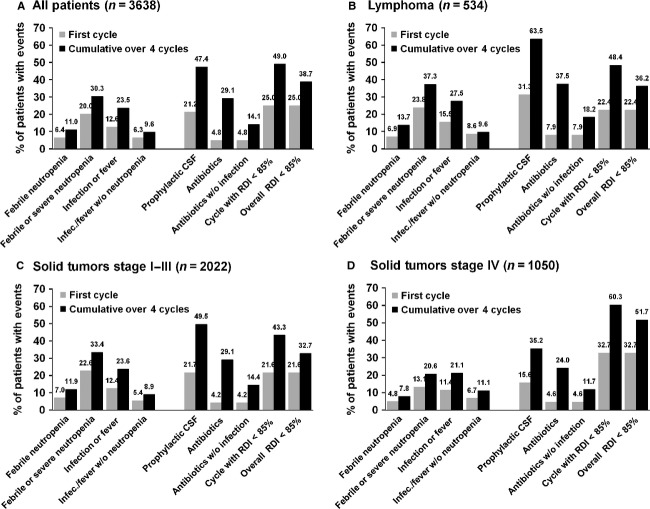
Neutropenic events versus supportive measures implemented. Data are presented for all patients together (A) and stratified by lymphoma (B), early stage (C), or late stage (D) solid tumor patients.

The majority of initial neutropenic and infection events occurred in cycle 1 (FN 6.4%, FN or SN 20%, infection or fever 12.6%) when compared to cumulative events over four cycles (FN 11%, FN or SN 30.3%, infection or fever 23.5%). This first cycle pattern was observed independently among early-stage, late-stage, and lymphoma patients (Fig. 3). The pattern was also observed irrespective of age group and also noted among patients who were receiving the first line of chemotherapy as well as those with prior history of chemotherapy. Patients with lymphoma and stage I–III solid tumors were less likely to undergo dose reductions and more likely to receive CSFs when compared to metastatic disease (Fig. 3). At the same time, for all patients, regardless of tumor type or stage, a substantial portion of supporting measures and chemotherapy dose reductions were added later in addition to those implemented at the initiation of the treatment.

### Completion of treatment

**Figure 4:**
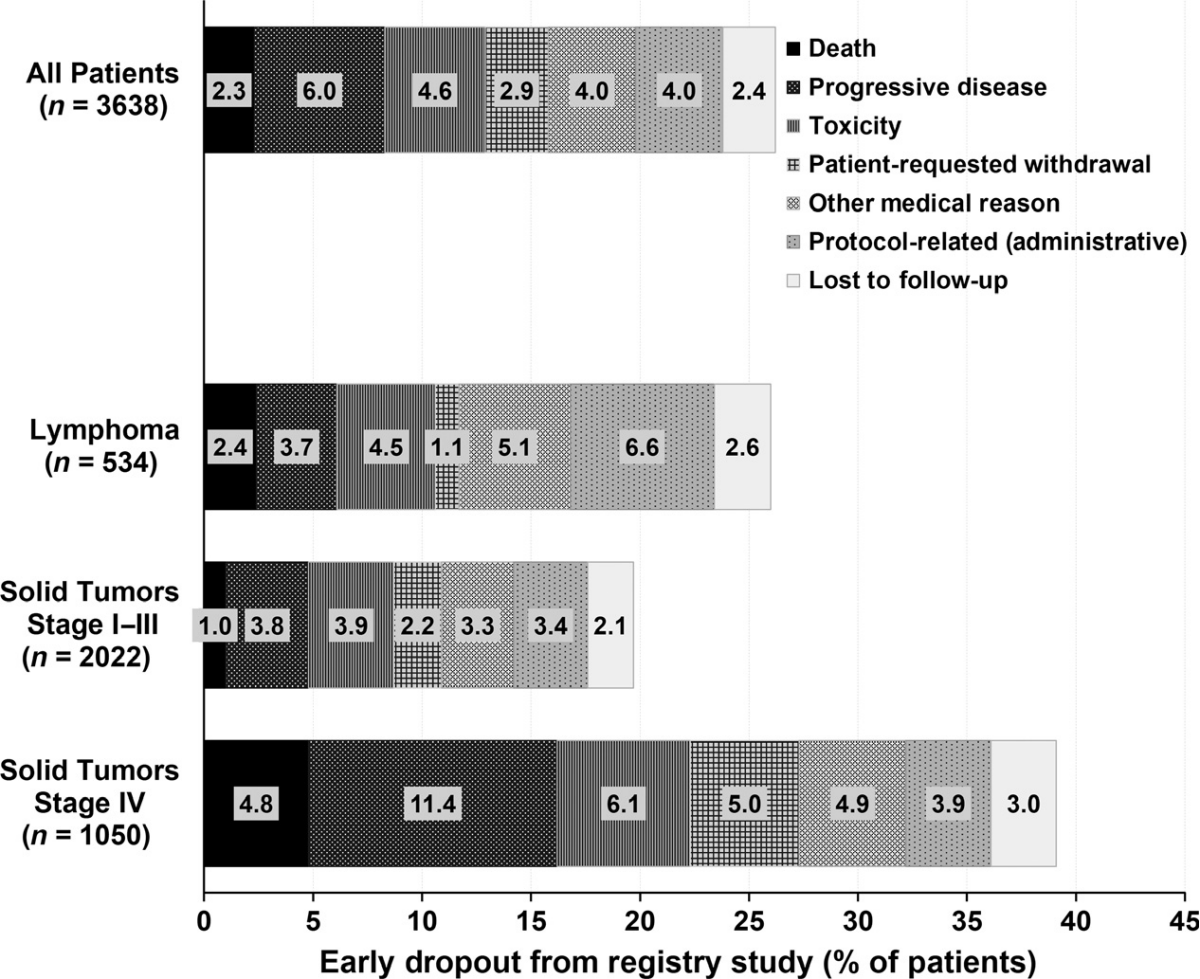
Reason for not completing full four cycles of chemotherapy.

**Figure 5:**
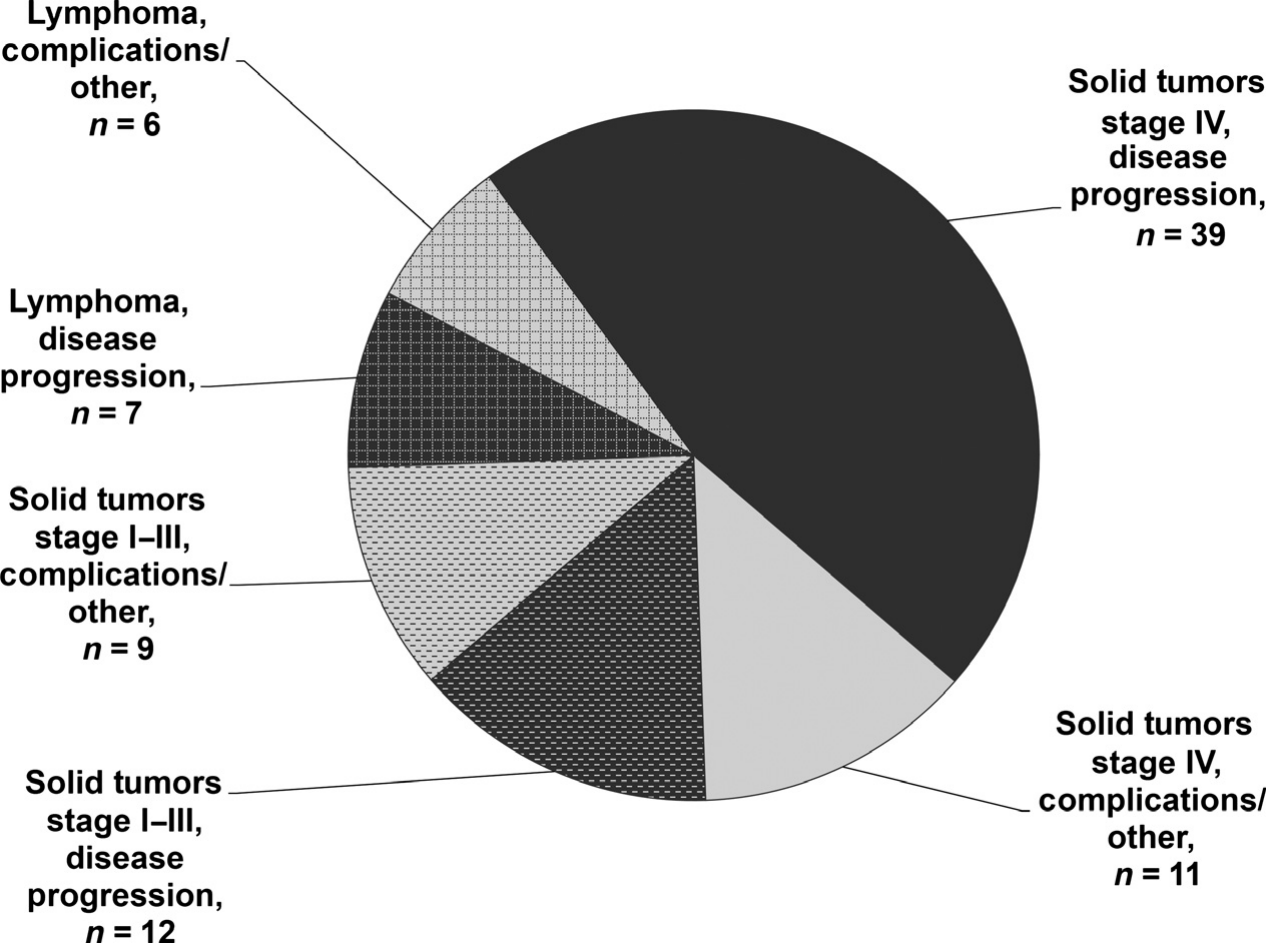
Early mortality events due to disease progression or other reasons such as treatment complications.

Of 3638 patients, 2681 (73.7%) completed four cycles of chemotherapy of which, 482 (18.0%) had missing toxicity data concerning the nadir of cycle 4. The reported reasons for not completing study in the 957 (26.3%) patients are depicted in Figure 4. Approximately 5% of all patients did not complete four cycles due to toxicity. A total of 84 patients (2.3%) died, 58 due to disease progression and 26 due to other reasons including treatment-related toxicity (Fig. 5). While the majority of deaths were due to disease progression in stage IV patients, deaths due to complications and causes other than progression contributed to almost half of deaths among lymphoma and early-stage patients.

## Discussion

Consistent with other studies 8–11,14, this large prospective cohort study demonstrated that the risk of neutropenic complications is greatest during the initial cycle of chemotherapy treatment. The reduction in risk of neutropenic complications including FN after the first cycle of chemotherapy appears to relate primarily to interventions implemented by medical providers in response to first cycle events. In the study by Aarts et al. 15, breast cancer patients with estimated risk of FN > 20% who were considered fit to receive 3-weekly polychemotherapy were randomized to primary granulocyte colony-stimulating factor (G-CSF) prophylaxis during the first two cycles (experimental arm) or to G-CSF prophylaxis during all cycles (standard arm). In the standard arm, 10% of patients had at least one FN event, whereas 36% of patients in the experimental arm experienced FN with the peak at the third cycle (24%), after G-CSF stopped.

In our study, we confirmed that clinicians' responses to early neutropenic events to reduce the risk of these complications, such as reduced chemotherapy DI and use of prophylactic CSFs or antibiotics, varied considerably with cancer type and stage. Also, the response appears to relate, in part, to whether the patient is being treated with curative intent (lymphoma; stage I–III solid tumors), where secondary use of CSFs is preferred, or whether the treatment is for advanced disease where reductions in RDI are more frequent. Prophylactic CSFs were employed to reduce the risk of neutropenic events or to sustain RDI in half to two-thirds of patients with lymphoma and early-stage solid tumors. In comparison, two-thirds of patients with advanced solid tumors had reduced RDI, which may relate to observed lower rates of neutropenic complications. Age and prior history of chemotherapy also seem to influence RDI with reductions <85% in more than half of the elderly (age ≥ 65) and among those with a prior history of chemotherapy.

RCTs and a meta-analysis of RCTs have demonstrated the efficacy and safety of primary prophylaxis with myeloid growth factors including significant reduction in the risk of FN, early mortality, and infection-related mortality in patients receiving chemotherapy 8,16,17. In elderly patients, the incidence of neutropenic events was reduced by nearly 60% with first cycle prophylactic growth factors use when compared to subsequent use after the initial event 18. Current clinical guidelines addressing the myeloid growth factors use recommend routine use of prophylactic CSFs to support chemotherapy in patients with 20% or greater risk of FN with lower risk regimens dependent upon patient-specific risk factors 19–21.

Approximately one-third of the patients with lymphoma or stage I–III solid tumors and more than half of patients with stage IV solid tumors had overall actual RDI < 85%. The impact of FN on chemotherapy dose delivery has been reviewed in the past 22. The importance of dose-dense chemotherapy in improving the clinical outcomes, especially in early-stage breast cancer, lymphoma, and small cell lung cancer, has also been extensively studied 23–25. Although RCTs have demonstrated the importance of maintaining chemotherapy DI in connection with improved survival in some settings 26,27, previous studies in patients with breast cancer and lymphoma have reported that approximately one-half of the patients receive substantial reductions in RDI 28,29.

The incidence of neutropenic events and fever or infection decreased in subsequent cycles of chemotherapy along with an increase in use of antibiotics, while nearly 15% of patients received antibiotics in the absence of reported fever or infection. The National Cancer Center Network (NCCN) guidelines recommend antibacterial prophylaxis if the ANC is expected to be less than 1000/mm^3^ for seven or more days 30. Recent American Society of Clinical Oncology (ASCO) guidelines recommend against routine use of antimicrobial prophylaxis in neutropenic patients unless ANC < 100/mm^3^ is expected for seven or more days or there are other factors that increase the risks for complications or mortality 31. As antibiotics do not decrease the risk of neutropenia and there are serious concerns about emerging antibiotics resistance, the routine use of prophylactic antibiotics as a support of chemotherapy is discouraged by ASCO, NCCN, and the European Organization for Research and Treatment of Cancer (EORTC), with exceptions for very high-risk settings such as acute leukemia and stem cell transplantation 19–21.

A limitation of the current study is a lack of specific information about the reasons for the treatment and supportive care decisions as well as lack of data on long-term outcomes. Likewise, information about the fourth cycle was not fully complete, which could lead to underestimation of events. Patients who dropped out from the study early might represent high-risk patients for toxicity as well as end-of-life care issues. Despite these limitations, this study is the largest prospective US cohort study tabulating chemotherapy-related toxicity patterns undertaken in a broad community oncology practice setting caring for a wide spectrum of patients including those with more complex medical conditions who are often excluded from clinical trials.

In conclusion, neutropenic events early in a course of chemotherapy are associated with chemotherapy dose reduction and treatment delays as well as increase in supportive care in an effort to reduce the risk of such events in subsequent cycles. There is evidence suggesting that the risk of neutropenic events after cycle 1 would not decrease if patients continued to receive full-dose chemotherapy without the addition of supportive treatment. Newly developed pretreatment risk assessment models, once fully validated, have the potential to help identify patients who are at high risk of neutropenic complications and therefore are likely to benefit from upfront prophylactic measures 32–35. The targeted use of preventive strategies in high-risk patients may help reduce the frequency of serious treatment-related complications as well as minimize unnecessary use of supportive care in low-risk patients, potentially leading to a reduction in health care costs and better quality of life for cancer patients receiving chemotherapy.

## Conflict of Interest

Duke University receives research grant support for the ANC Study Group Coordinating Center from Amgen (Dr. Lyman, PI). Dr. Dale receives research support from Amgen.
